# Papel do Sistema Nervoso Autônomo na Fibrilação Atrial

**DOI:** 10.36660/abc.20210771

**Published:** 2021-11-01

**Affiliations:** 1 Hospital do Coração São Paulo SP Brasil Hospital do Coração, São Paulo, SP – Brasil

**Keywords:** Sistema Nervoso Autônomo, Fibrilação Atrial, Cardioneuroablação, Sistema Nervoso Cardíaco

Desde o final dos anos 90 estamos estudando profundamente o papel do sistema nervoso autônomo na gênese e manutenção da fibrilação atrial (FA) e da síncope neurocardiogênica.^1^

A FA pode ocorrer tanto em corações normais, como naqueles com importantes alterações estruturais.^2^ Episódios de FA em indivíduos jovens e com corações aparentemente normais sempre nos chamaram a atenção. O fato desses pacientes terem frequentemente manifestações do tipo disautonômicas, nos levou a pensar que a denervação proposital poderia ser uma forma de tratamento desses casos sendo esta técnica pioneiramente publicada em 2004.^3^ O efeito da inervação não parece apenas ser devido ao estímulo neural, mas também por promover desordem na arquitetura sincicial atrial, nos locais da penetração dos neurônios, que afastam as células miocárdicas, desconectando-as. Esse desarranjo promove também alterações que podem ser detectadas no endocárdio pelo sinal elétrico local, o que chamamos Ninhos de FA (NFA), e no caso da inervação, chamamos de NFA tipo I.

Os NFA fazem parte do substrato da FA, porém, para que seja sustentada, é necessário a existência de um mantenedor que chamamos de taquicardia de Background (TBK).^4^ A eliminação da FA parece depender da eliminação do substrato e do fator mantenedor, de acordo com nossas observações e também conforme achados de vários autores mais recentemente.^5^ A estimulação vagal reduz de forma importante o período refratário das células cardíacas, favorecendo a indução da FA.^6^ O mais interessante é que essa alteração do período refratário não é homogênea, o que facilita mais a arritmia.

Outro dado importante é que as regiões inervadas pelo vago direito e esquerdo, embora parcialmente superponíveis, não são exatamente as mesmas.^7^ Além disso, a estimulação dos gânglios estrelados ou a infusão de simpaticomiméticos associadas à estimulação vagal reduz significativamente o limiar de FA dos pacientes avaliados, mostrando que o equilíbrio entre o sistema simpático e parassimpático é primordial para manutenção do ritmo cardíaco regular. Os autores deste estudo também encontraram dados semelhantes ao estudar tanto a inervação intrínseca cardíaca como seus receptores.^8^ O trabalho está muito bem conduzido, selecionando 2 grupos muito semelhantes e com as mesmas patologias, nos quais a diferença é que apenas um grupo apresentava FA. Os autores tiveram o cuidado de ajustar as variáveis com ferramentas estatísticas, o tamanho do átrio esquerdo e o tempo mínimo de diagnóstico, para evitar interferência nos resultados. As amostras também foram coletadas em regiões próximas aos principais gânglios paracardíacos e, portanto, locais mais densamente inervados e previamente relacionados à FA.^9^ Além disso, não somente os nervos, mas também os diversos receptores foram avaliados. O aumento de inervação simpática nessas regiões nos portadores de FA corrobora dados da literatura, mas ainda não tem explicação adequada que a justifique. O desequilíbrio da inervação simpática/parassimpática certamente é arritmogênico, como já foi extensamente descrito.^10^ É possível que isso comprove a eficiência do uso de beta-bloqueadores no tratamento de muitos casos de FA, recuperando o equilíbrio entre os dois sistemas, embora o número de receptores não foi modificado pelo uso desta medicação como observado neste trabalho.

Ficou claro, com os dados obtidos neste artigo, que a inervação intrínseca cardíaca tem papel primordial na manutenção da FA, independentemente da cardiopatia estrutural, e o tratamento desta arritmia deve incluir a abordagem do sistema nervoso autônomo, quer seja através do uso drogas ou mesmo através da ablação destas regiões mais densamente inervadas, para que possamos ter resultados mais robustos e duradouros. Desta forma, consideramos fundamental insistir profundamente na pesquisa do sistema nervoso autônomo do coração como é descrito neste artigo, para entendimento da gênese dos diversos tipos de FA, assim como para identificar diferentes formas de tratamento desta arritmia tão prevalente atualmente na população geral.

## References

[1] Pachon MJC, Pachon MEI, Pachon MJC, Lobo TJ, Pachon MZ, Vargas RN, et al. A New Treatment for Atrial Fibrillation Based on Spectral Analysis to Guide the Catheter RF-Ablation. Europace. 2004;6(6):590-601. doi: 10.1016/j.eupc.2004.08.005. 10.1016/j.eupc.2004.08.005 15519263

[2] Cintra FD, Figueiredo MJO. Atrial Fibrillation (Part 1): Pathophysiology, Risk Factors, and Therapeutic Basis. Arq Bras Cardiol. 2021;116(1):129-39. doi: 10.36660/abc.20200485. 10.36660/abc.20200485 PMC8159512 33566977

[3] Pachon JC, Pachon EI, Pachon JC, Lobo TJ, Pachon MZ, Vargas RN, et al. “Cardioneuroablation”––New Treatment for Neurocardiogenic Syncope, Functional AV Block and Sinus Dysfunction Using Catheter RF-Ablation. Europace. 2005;7(1):1-13. doi: 10.1016/j.eupc.2004.10.003. 10.1016/j.eupc.2004.10.003 15670960

[4] Pachón-M JC, Pachón-M EI, Santillana P TG, Lobo TJ, Pachón CTC, Pachón-M JC, et al. Ablation of “Background Tachycardia” in Long Standing Atrial Fibrillation: Improving the Outcomes by Unmasking a Residual Atrial Fibrillation Perpetuator. J Atr Fibrillation. 2017;10(2):1583. doi: 10.4022/jafib.1583. 10.4022/jafib.1583 PMC5673289 29250230

[5] Oketani N, Lockwood E, Nademanee K. Incidence and Mode of AF Termination During Substrate Ablation of AF Guided Solely by Complex Fractionated Atrial Electrogram Mapping. Circulation 2008;118(Suppl 18):925. doi: 10.1161/circ.118.suppl_18.S_925-a.

[6] Pachon M JC, Pachon M EI, Santillana P TG, Lobo TJ, Pachon CTC, Pachon M JC, et al. Simplified Method for Vagal Effect Evaluation in Cardiac Ablation and Electrophysiological Procedures. JACC Clin Electrophysiol. 2015;1(5):451-60. doi: 10.1016/j.jacep.2015.06.008. 10.1016/j.jacep.2015.06.008 29759475

[7] Pachon-M EI, Pachon-Mateos JC, Higuti C, Santillana-P TG, Lobo T, Pachon C, et al. Relation of Fractionated Atrial Potentials with the Vagal Innervation Evaluated by Extracardiac Vagal Stimulation During Cardioneuroablation. Circ Arrhythm Electrophysiol. 2020;13(4):e007900. doi: 10.1161/CIRCEP.119.007900. 10.1161/CIRCEP.119.007900 32188285

[8] Oliveira IM, Silva EL Jr, Martins YO, Rocha HAL, Scanavacca MI, Gutierrez PS. A Remodelação do Sistema Nervoso Autônomo Cardíaco pode Desempenhar um Papel na Fibrilação Atrial: Um Estudo do Sistema Nervoso Autônomo e Receptores Miocárdicos. Arq Bras Cardiol. 2021; 117(5):999-1007. 10.36660/abc.20200725 PMC8682090 34406322

[9] Driessen AHG, Berger WR, Krul SPJ, van den Berg NWE, Neefs J, Piersma FR, et al. Ganglion Plexus Ablation in Advanced Atrial Fibrillation: The AFACT Study. J Am Coll Cardiol. 2016;68(11):1155-65. doi: 10.1016/j.jacc.2016.06.036. 10.1016/j.jacc.2016.06.036 27609676

[10] Xi Y, Cheng J. Dysfunction of the Autonomic Nervous System in Atrial Fibrillation. J Thorac Dis. 2015;7(2):193-8. doi: 10.3978/j.issn.2072-1439.2015.01.12. 10.3978/j.issn.2072-1439.2015.01.12 PMC4321068 25713736

