# Supplementary material for: Papel do Sistema Nervoso Autônomo na Fibrilação Atrial
Source: Arq Bras Cardiol. 2021 Nov 1;117(5):1008–9. [Article in Portuguese] doi: 10.36660/abc.20210771 (PMC8682107; doi:10.36660/abc.20210771)
Supplement: Translation

# Translation: Role of the Autonomic Nervous System in Atrial Fibrillation

**Affiliations:** 1 São Paulo SP Brazil Hospital do Coração, São Paulo, SP – Brazil

**Keywords:** Autonomic Nervous System, Atrial Fibrillation, Cardioneuroablation, Cardiac Nervous System

Since the late 1990s, we have been intensely studying the role of the autonomic nervous system in the genesis and maintenance of atrial fibrillation (AF) and neurocardiogenic syncope.^1^

AF can occur both in normal hearts and in those with significant structural changes.^2^ Episodes of AF in young individuals with apparently normal heart have always caught our attention. The fact that these patients often have dysautonomic-like manifestations led us to think that purposeful denervation could be a way to treat these cases, and this technique was pioneered in 2004.^3^ The effect of innervation seems to be not only due to neural stimulation but also by promoting disarray in the atrial syncytial architecture, in the sites of penetration of neurons, which detach the myocardial cells, disconnecting them. This disarrangement also promotes changes that can be detected in the endocardium by the local electrical signal, which we call AF Nests (AFN), and in the case of innervation, we call AFN type I.

The AFN are part of the AF substrate but to be sustained, it is necessary to have a maintainer that we call background tachycardia (BKT).^4^ The AF elimination seems to depend on the elimination of substrate and sustaining factor, according to our observations and the findings of several more recent authors.^5^ The vagal stimulation significantly reduces the refractory period of cardiac cells, facilitating the induction of AF.^6^ The most interesting is that this alteration in the refractory period is not homogeneous, favoring arrhythmia.

Another important fact is that the regions innervated by the right and left vagus, although partially overlapping, are not precisely the same.^7^ Furthermore, the stimulation of the stellate ganglia or the infusion of sympathomimetics associated with vagal stimulation significantly reduces the AF threshold of the evaluated patients, showing that the balance between the sympathetic and parasympathetic system is primordial for the maintenance of regular cardiac rhythm. The authors of this study also found similar data when studying both the intrinsic cardiac innervation and its receptors.^8^ The work is very well conducted, selecting two very similar groups with the same pathologies, in which the difference is that only one group had AF. The authors were careful to adjust the variables with statistical tools, the size of the left atrium, and the minimum diagnostic time to avoid interference in the results. The samples were also collected in regions close to the main paracardiac ganglia, and therefore sites more densely innervated and previously related to AF.^9^ In addition, not only nerves but also the various receptors were evaluated. The increased sympathetic innervation in these regions in AF patients corroborates data from the literature, but there is still no adequate explanation to justify it. The unbalance of sympathetic/parasympathetic innervation is certainly arrhythmogenic, as already extensively described.^10^ It is possible that this proves the efficiency of the use of beta-blockers in the treatment of many cases of AF, restoring the balance between the two systems, although the number of receptors was not modified by the use of this medication as observed in this paper.

It was clear from the data obtained in this article that intrinsic cardiac innervation plays a crucial role in maintaining AF, regardless of structural heart disease. The treatment of this arrhythmia should include the approach of the autonomic nervous system, whether through the use of drugs or even through the ablation of these more densely innervated regions so that we can have more robust and lasting results. In this way, we consider it fundamental to insist deeply in the research of the autonomic nervous system of the heart as described in this article, for understanding the genesis of the diverse types of AF, as well as to identify different forms of treatment of this arrhythmia that is so prevalent today in the general population.
